# Acute inhibition of PMCA4, but not global ablation, reduces blood pressure and arterial contractility via a nNOS‐dependent mechanism

**DOI:** 10.1111/jcmm.13371

**Published:** 2017-11-30

**Authors:** Sophronia Lewis, Robert Little, Florence Baudoin, Sukhpal Prehar, Ludwig Neyses, Elizabeth J. Cartwright, Clare Austin

**Affiliations:** ^1^ Faculty of Biology, Medicine and Health Division of Cardiovascular Sciences The University of Manchester, Manchester Academic Health Science Centre Manchester UK; ^2^ Present address: Centre for Cardiovascular Sciences Queen's Medical Research Institute University of Edinburgh Edinburgh EH16 4TJ UK; ^3^ Present address: University of Luxembourg Maison du Savoir 2, Avenue de l, Université Esch‐sur‐Alzette L‐4365 UK; ^4^ Present address: Faculty of Health and Social Care Edge Hill University St Helens Road Ormskirk Lancashire L39 4QP UK

**Keywords:** plasma membrane calcium ATPase, PMCA4, blood pressure, calcium, neuronal nitric oxide synthase, cellular, physiology, membrane, contractility

## Abstract

Cardiovascular disease is the world's leading cause of morbidity and mortality, with high blood pressure (BP) contributing to increased severity and number of adverse outcomes. Plasma membrane calcium ATPase 4 (PMCA4) has been previously shown to modulate systemic BP. However, published data are conflicting, with both overexpression and inhibition of PMCA4 *in vivo* shown to increase arterial contractility. Hence, our objective was to determine the role of PMCA4 in the regulation of BP and to further understand how PMCA4 functionally regulates BP using a novel specific inhibitor to PMCA4, aurintricarboxylic acid (ATA). Our approach assessed conscious BP and contractility of resistance arteries from PMCA4 global knockout (PMCA4KO) mice compared to wild‐type animals. Global ablation of PMCA4 had no significant effect on BP, arterial structure or isolated arterial contractility. ATA treatment significantly reduced BP and arterial contractility in wild‐type mice but had no significant effect in PMCA4KO mice. The effect of ATA *in vivo* and *ex vivo* was abolished by the neuronal nitric oxide synthase (nNOS) inhibitor Vinyl‐l‐NIO. Thus, this highlights differences in the effects of PMCA4 ablation and acute inhibition on the vasculature. Importantly, for doses here used, we show the vascular effects of ATA to be specific for PMCA4 and that ATA may be a further experimental tool for elucidating the role of PMCA4.

## Introduction

Cardiovascular diseases are the world's leading cause of morbidity and mortality, responsible for approximately 17 million deaths per annum 1, 2. Multiple causal factors can induce and promote cardiovascular disease, with elevated blood pressure (BP) being a highly significant factor for increased risk for heart disease and stroke 2, 3. In turn, understanding how BP is regulated may contribute to the development of novel treatment strategies for cardiovascular diseases, a particularly important requirement due to increased health burdens of an increasingly ageing population 4, 5.

Increased peripheral vascular resistance has been reported as the principal driver for chronically elevated BP 6. Small diameter arteries, termed resistance arteries, provide the greatest contribution to total peripheral vascular resistance 6, 7. Alterations in both the structure and contractile function of resistance arteries have been associated with increased BP 8, 9, 10. At the molecular level, the intracellular Ca^2+^ concentration ([Ca^2+^]_i_) and calcium signalling within vascular cells have long been known to be important for arterial function 11, 12. We have previously proposed that the ATP‐driven calmodulin‐dependent Ca^2+^ pump plasma membrane calcium ATPase 4 (PMCA4) has a role in arterial calcium handling 13, 14.

PMCA4 is present in vascular smooth muscle (VSM) of larger arteries 15, 16, and at the mRNA level has been proposed to be the predominant PMCA isoform in such tissue. Quantification of PMCA4 expression in small resistance arteries has proved challenging; hence, functional approaches have previously been employed. Initial studies employed mice overexpressing PMCA4 specifically in arterial VSM. These mice were shown to exhibit elevated unconscious BP, and small arteries excised from these animals showed increased contractility to different stimuli 14, 17. However, acute application of caloxin peptides 1b1 and 1c2, reported to inhibit PMCA4, also increased contractility of isolated arteries 18, 19. Whilst the seemingly conflicting observations from these studies may, in part, be attributable to different arterial beds being functionally studied, the effect of PMCA4 on resistance arterial contractility and BP remains poorly understood. Importantly, systemic BP and arterial contractility when PMCA4 is inhibited remain to be determined.

PMCAs are classically described as playing a role in calcium extrusion from cells, although it is now evident that they, and specifically PMCA4, also have important roles as scaffolding proteins and in signal transduction 13, 20, 21, 22, 23. PMCA4 has been shown to negatively regulate neuronal nitric oxide synthase (nNOS)‐mediated nitric oxide production in cellular assays 21 and to regulate cardiac signal transduction pathways *via* physical interaction with nNOS 20, 23. Hence, the increased arterial contractility observed with overexpression of PMCA4 may be attributed to negative regulatory effects of PMCA4 on nNOS vascular activity 14, 17. We sought to investigate this *in vivo* in our model.

In this study, we investigated, for the first time, the effects of PMCA4 ablation on BP and resistance arterial contractile function and, furthermore, examined the acute effects of a recently identified and validated inhibitor of PMCA4 24, 25 on these parameters.

## Materials and Methods

### Animals

The effect of global ablation of PMCA4 was assessed using 3‐month‐old PMCA4 germline‐null mutant adult male mice (PMCA4 knockout, PMCA4KO) which we have previously generated 26. In all experiments, the phenotype and vascular function of male PMCA4KO mice were compared to male wild‐type littermate controls (PMCA4WT) on a mixed C57Bl/6J/129Sv background 26. To investigate the effect of pharmacological inhibition of PMCA4, male wild‐type (WT) mice of a 129Sv background were used. Mice were maintained in a pathogen‐free facility and housed under a 12‐hour light/dark cycle with *ad libitum* access to normal chow diet and water. All experiments were approved by the University of Manchester Ethics Committee and were in accordance with the United Kingdom Animals (Scientific Procedures) Act 1986. All animals were humanely killed by cervical dislocation. This study conforms to ARRIVE guidelines on use of experimental animals 27.

### Conscious blood pressure recording

*In vivo* conscious BP of mice was monitored using a CODA™ occlusion tail cuff volume–pressure sensor monitoring system (Kent Scientific Corporation, Torrington, Connecticut, USA). Mice were acclimatized to the animal holder and the system for three consecutive days prior to experimental recording. For experimental recordings, mice were placed on a pad heated to 37°C and blood flow to the distal tail was occluded with a maximal cuffing pressure of 250 mmHg and then steadily deflated over 15 sec. for a single cycle. Systolic and diastolic blood pressures were automatically recorded during cuff deflation as blood flowed into the tail. Twenty continuous cycles were performed (10‐min experiment), with accepted values (blood volume returning through cuff being ≥15 μl in calm and relaxed animals) from the latter 10 cycles used for data analysis. Five seconds between each cycle was programmed. Basal BP of PMCA4KO mice was compared to wild‐type littermates (PMCA4WT). In separate experiments, short‐term effects of a recently identified and characterized inhibitor of PMCA4, aurintricarboxylic acid (ATA), were examined in WT and PMCA4KO mice 24, 25. The effects of ATA (5 mg/kg bodyweight) on conscious BP were examined 90 min. after intraperitoneal injection (i.p.) and were compared to vehicle (50% DMSO, 50% sterile water, v/v)‐injected mice (animals randomly assigned treatment or vehicle). We have previously shown a comparable dose of ATA *in vitro* to completely inhibit PMCA4, and *in vivo*, this dose reverses cardiac hypertrophy 24, 25. Further, the mechanism of action of ATA on BP was assessed by injecting the nNOS inhibitor Vinyl‐l‐NIO (VLN) at 10 mg/kg bodyweight, a dose comparable to a concentration exceeding the Ki value for inhibition of nNOS 28. Basal BP of PMCA4WT mice was compared to animals injected i.p. with vehicle (25% DMSO, 75% sterile water, v/v) and ATA (5 mg/kg bodyweight 5 min. later), to animals injected with VLN (10 mg/kg bodyweight) and then vehicle (50% DMSO, 50% sterile water, v/v) and to animals injected with VLN (10 mg/kg bodyweight) 5 min. preceding injection of ATA (5 mg/kg bodyweight). All BP experiments were performed between 09:00 and 12:00 hrs.

### Dissection of tissues

The entire mesenteric bed and thoracic aorta were removed and placed separately into ice‐cold physiological salt solution (PSS) of the following composition: NaCl 119 mM, KCl 4.7 mM, MgSO_4_.7H_2_O 1.17 mM, NaHCO_3_ 25 mM, KH_2_PO_4_ 1.17 mM, K_2_EDTA 0.03 mM, glucose 5.5 mM and CaCl_2_.2H_2_O 1.6 mM (pH 7.4, 95% air/5% CO_2_). Fat and adherent tissue was removed from third‐order mesenteric arteries and aorta. Vascular smooth muscle cells were immediately isolated from mesenteric arteries, or arteries were used for pressure myography studies. Aortic tissue was fixed for immunohistochemical analysis or frozen in liquid nitrogen before being stored at −80°C until proteins were extracted from the tissue.

### Vascular smooth muscle cell (VSMC) isolation

Third‐order mesenteric arteries (*n* = 3 animals as separate experiments) were cleared of attached fat and incubated in HEPES‐buffered physiological solution (NaCl 154 mM, KCl 5.4 mM, MgSO_4_ 1.2 mM, glucose 10 mM, CaCl_2_ 10 mM and HEPES 10 mM; pH 7.4) supplemented with 1,4‐dithioerythritol (Sigma‐Aldrich, Gillingham, UK) at 2 mg/ml and papain (Worthington Biochemical, Lakewood, New Jersey, USA) at 1 mg/ml in a water bath at 37°C for 22 min. The solution was then replaced with HEPES buffer supplemented with type F collagenase from Clostridium histolyticum (Sigma‐Aldrich, Gillingham, UK) at 1 mg/ml for 7 min. The cell suspension was briefly stood on ice before being gently titrated and small drops of cell suspension pipetted onto poly‐l‐lysine‐coated slides (VWR International, Radnor, Pennsylvania, USA). Slides were left at room temperature for 50 min. before ice‐cold methanol was added to the cell suspension for 10 min. Slides were carefully washed in HEPES buffer before being stored under saline solution at 4°C before being probed for PMCA4 as described below.

### Immunohistochemistry

Aortas were segmented to approximately 1.5 cm in length, immersed in excess 4% paraformaldehyde (in 0.1 M PBS) for 6 hrs, serially dehydrated in increasing concentrations of ethanol and paraffin‐embedded. Five‐micrometre‐thick cross sections were cut, mounted onto poly‐l‐lysine‐coated slides (VWR International, Radnor, Pennsylvanis, USA) and dried overnight at 37°C. Sections were incubated in 3% hydrogen peroxide and washed in double‐distilled water followed by phosphate buffered saline (PBS) before incubation in proteinase K in PBS (0.1 mg/ml). Sections were permeabilized with Triton X‐100 (BDH Ltd, Poole, UK) in PBS (0.1%) for 10 min. and washed in PBS. To block non‐specific binding, sections were incubated with 3% bovine serum albumin (BSA) in PBS for an hour and washed in PBS. Isolated VSMCs were incubated under 10% donkey serum in PBS (v/v) for 2 hrs at laboratory temperature. Sections or cells were probed with primary antibody for α‐smooth muscle actin (αSMA) (1:100; Abcam, Cambridge, Massachusetts, USA), the endothelial cell marker CD34 (1:50; Abcam, Cambridge, Massachusetts, USA) or PMCA4 (1:50; Abcam, Cambridge, Massachusetts, USA) in blocking solution and incubated overnight at 4°C. Slides were washed three times with PBS and then incubated with secondary antibody conjugated with Texas Red (red) or FITC (green) fluorescent labels (Cell Signaling Technology, Danvers, Massachusetts, USA) for 2 hrs at room temperature. Slides were washed three times in PBS before being stained with 3 μM of 4′,6‐diamidino‐2‐phenylindole dihydrochloride (DAPI) (diluted 1:5000 in PBS; Invitrogen Ltd, Carlsbad, California, USA) for 60 sec., washed in PBS and mounted under coverslips using mounting medium (Vectashield; Vector Laboratories, Peterborough, UK). Sections were visualized using an upright confocal fluorescence microscope (Nikon, Eclipse C1 Plus, using oil objectives) with Texas Red, FITC green and blue filters using the EZ‐C1 3.90 FreeViewer software (Nikon Corporation, Nikon UK Ltd, Surrey UK). VSMCs were imaged with a Leica DM5000B microscope and Leica DFC 3000 g camera, and using Leica 10X capture software (Leica Microsystems, Milton Keynes, UK).

### Western blotting

Aortic tissue was homogenized and proteins isolated and transferred as previously described 29. Blots were blocked in 3% milk in PBS with 0.05% Tween‐20 and hybridized to primary antibodies: rabbit anti‐PMCA1 (to 1 μg/ml, Abcam, expected molecular weight 110 kD) and rabbit anti‐NCX1 (sodium–calcium exchanger, to 5 μg/ml, Santa Cruz, (Dallas, Texas, USA) expected molecular weight about 120 kD), respectively; and subsequently to a secondary anti‐rabbit HRP‐coupled antibody (1:2000 dilution; Dako (Stockport, UK) and Cell Signaling (Danvers, Massachusetts, USA)). Blots were probed for β‐actin (Abcam, molecular weight 42 kD) and subsequently incubated in 1.5% glycine (with 1% Tween‐20 and 0.1% SDS) at room temperature for 60 min. and then reprobed for α‐tubulin (Abcam, molecular weight 50 kD) as a loading control. All proteins were detected with enhanced chemiluminescent solution (Fisher Scientific, Loughborough, UK) in the dark and visualized by a chemiluminescence detection system (Molecular Imager ChemiDoc™ XRS; Bio‐Rad, Hemel Hempstead, UK). Results of protein band intensities were quantified using ImageJ software relative to the loaded level of α‐tubulin.

### Pressure myography

Small mesenteric arterial segments of 2–3 mm length were mounted on a pressure myograph (Living Systems Instrumentation, St Albans, Vermont, USA) as previously described 30, 31, 32. Arteries were pressurized to an intravascular pressure of 60 mmHg, superfused with PSS (37°C, 95% air/5% CO_2_) and left to equilibrate for 30 min. Contractile responses to 100 mM potassium solution (KPSS) (potassium being isosmotically substituted for sodium) and to cumulative doses of norepinephrine (NE) (1 nM–30 μM) were determined. To assess the effects of acute PMCA4 inhibition on contractile responses, experiments were performed in the presence of 1 μM or 10 μM ATA or 100 μM caloxin 1b1 following an initial 30‐min perfusion of the inhibitor. Such concentrations were chosen based on our previous reports that 1 μM ATA completely blocked PMCA4 activity in membrane microsomes derived from HEK293 cells overexpressing PMCA4 whilst having no effect on Na^+^/K^+^ ATPase activity and little effect on PMCA1 activity 24. At 10 μM, we have shown that ATA has no significant effect on global calcium transients in adult cardiomyocytes 24. Caloxin 1b1 at 100 μM has been shown to have maximal functional effects in isolated arterial preparations 18. Further experiments were performed in the presence of the non‐specific NOS inhibitor N‐ω‐nitro‐l‐arginine (LNNA) (100 μM, 30 min. of incubation) 30 or the specific nNOS inhibitor Vinyl‐l‐NIO (VLN) (10 μM, 30 min. of incubation) 33, doses selected to maximally inhibit enzymatic activity 28, 34. Arterial passive properties were determined following 15 min. of perfusion of Ca^2+^ chelated PSS solution (Ca^2+^‐free) with graded increases in intravascular pressure from 5 to 140 mmHg, as previously described 30, 31, 32.

For simultaneous measurement of intracellular free calcium ([Ca^2+^]_i_) and arterial contractility, isolated segments of mesenteric arteries were incubated with 20 μM Indo‐1‐AM (Cell Signaling Technology, Danvers, Massachusetts, USA) in HEPES‐buffered physiological solution for 2 hrs. Arteries were mounted in the bath of a pressure myograph placed atop an inverted microscope, excited at 340 nm and emissions measured *via* photomultipliers at 400 and 500 nm. The 400 nm:500 nm emission ratio (F_400_/F_500_) was determined (following correction for autofluorescence) and used as an indicator of [Ca ^2+^]_i_, as previously described 35.

### Analysis

Results are expressed as mean ± S.E.M. (standard error mean) for the number of animals (*n*) used. Contractile responses are expressed as a percentage of the resting lumen diameter. From dose–response curves to NE, the maximal response (Rmax) and EC_50_ value that induced half‐maximal constriction were calculated. Measurements of internal lumen diameter and wall thickness in Ca^2+^‐free solution were used to calculate structural passive properties, as previously described 36. The differences between means were considered significant at *P* < 0.05 (*). Dose–response curves were analysed by two‐way analysis of variance (anova) for repeated measures followed by the Bonferroni post hoc test if *P* < 0.05. Statistical comparisons of all other data were performed using an unpaired Student's *t*‐test. All statistical evaluations were performed using GraphPad Prism software (GraphPad Software, La Jolla, California, USA).

## Results

### Expression of PMCA4

**Figure 1:**
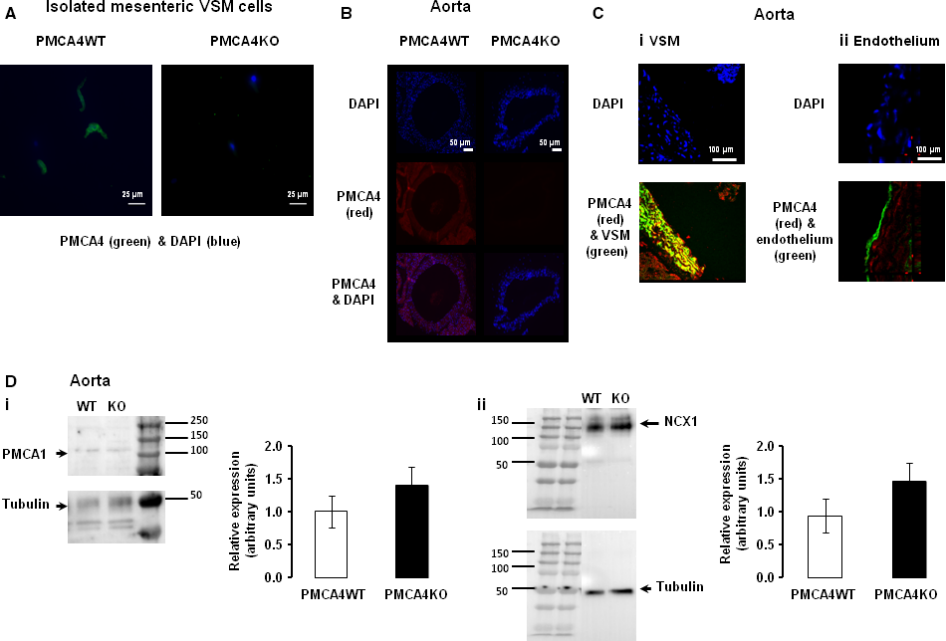
PMCA4 protein is absent from vascular cells and tissues of PMCA4KO mice, but expression of other calcium handling proteins was not significantly altered in this model. PMCA4 protein could not be detected in isolated VSMCs (**A**) and aorta (**B**) from PMCA4KO mice by immunohistochemistry (representative images from three experiments). PMCA4 protein was principally detected in VSMCs, but not in the endothelium of aorta from PMCA4WT mice (**C**) (representative images from 3 experiments). Nuclei stained with DAPI shown as blue in images. Western blot analysis showed aortic protein expression of PMCA1 and NCX1 was similar in PMCA4WT and PMCA4KO mice (**D**; mean ± S.E.M., *n* = 6 and 6, and 5 and 5, respectively).

PMCA4 expression has been shown to be predominant in VSM but not in vascular endothelial cells 15, 16. In the present study, we showed that VSMCs isolated from mesenteric arteries and aortic segments from PMCA4KO mice were completely devoid of PMCA4 protein as determined by immunohistochemistry (Fig. 1A and B). In fixed aortic tissue from PMCA4WT mice, PMCA4 protein was localized predominantly to VSMCs with little expression being evident in endothelial cells (Fig. 1C). Aortic protein expression of PMCA1 and the sodium–calcium exchanger 1 (NCX1) was unaffected by ablation of PMCA4 (Fig. 1D).

### *In vivo* conscious BP

**Figure 2:**
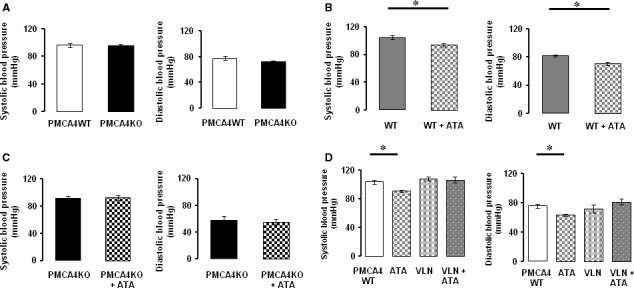
Differential effect of ablation and inhibition of PMCA4 on basal conscious blood pressure. Conscious systolic blood pressure and diastolic blood pressure (BP) were not significantly altered by ablation of PMCA4 (**A**; mean ± S.E.M., *n* = 6 and 6); however, ATA (BP recorded 90 min. after 5 mg/kg i.p. injection) significantly (*P < 0.05) reduced conscious BP in WT mice (**B**; mean ± S.E.M., *n* = 5 and 6). ATA treatment did not significantly affect conscious BP of PMCA4KO mice (**C**; mean ± S.E.M., *n* = 5 and 5). In PMCA4WT mice, ATA treatment significantly (*P < 0.05) reduced systolic BP; however, no reduction in BP was observed following treatment with the specific nNOS inhibitor, Vinyl‐l‐NIO (VLN), alone or with both ATA and VLN (**D**; mean ± S.E.M., *n* = 11, 3, 3 and 5).

Ablation of PMCA4 had no effect on basal systolic and diastolic BP (Fig. 2A); however, 90 min. after injection with the PMCA4 inhibitor ATA (5 mg/kg), a significant reduction in both systolic BP (104 ± 3 mmHg to 94 ± 2 mmHg) and diastolic BP (82 ± 1 mmHg to 70 ± 2 mmHg) was recorded in WT mice (Fig. 2B). Such findings were found to be replicable on separate experimental days (data not shown). The spread of BP recordings for each experimental group (as shown by S.E.M. and in Fig. S1) was low, and as such, small changes in the absolute level of BP could be detected.

In PMCA4WT mice, ATA had no significant effect on BP in the presence of the nNOS inhibitor VLN (Fig. 2D). Importantly, when PMCA4KO mice were treated with ATA, no significant effect on systolic and on diastolic BP was detected (Fig. 2C). Pulse rate did not significantly differ between PMCA4WT and PMCA4KO mice (733 ± 17 bpm and 692 ± 26 bpm, respectively, data not shown), or between WT mice injected with vehicle or ATA (735 ± 18 bpm and 768 ± 15 bpm for vehicle and ATA treatment, respectively, data not shown). Administration of vehicle had no significant effect on systolic BP (103 ± 4 mmHg basal and 104 ± 3 mmHg) and on diastolic BP (75 ± 3 mmHg basal and 82 ± 1 mmHg) of WT animals 90 min. after injection.

### Isolated arterial function

**Figure 3:**
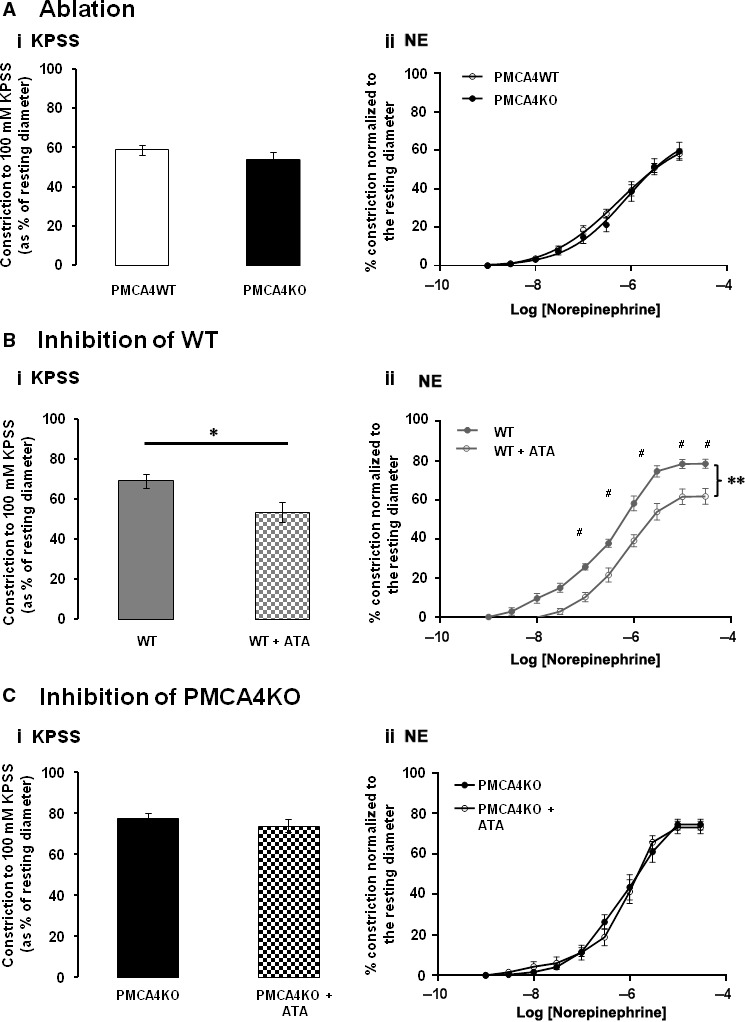
Ablation and inhibition of PMCA4 have different effects on mesenteric artery constriction. The magnitude of arterial contraction in response to KPSS (100 mM K^+^) (**Ai**) and cumulative dose response to norepinephrine (NE) (**Aii**) is not significantly different between PMCA4KO and PMCA4WT arteries. Mean ± S.E.M., *n* = 11 and 14. In WT mice, ATA (10 μM) significantly reduced the magnitude of arterial contraction in response to KPSS (**B**i; *P < 0.05, Student's *t*‐test) and the cumulative dose response to NE (**B**ii; **P < 0.05 repeated‐measures ANOVA). Bonferroni post‐test analysis revealed significant reduction with ATA at higher doses of NE (#*P* < 0.05). Mean ± S.E.M., *n* = 8 and 8. 10 μM ATA does not have a significant effect on the magnitude of arterial contraction in response to KPSS (**Ci**;* t*‐test) or on the cumulative dose response to NE (**Cii**; repeated‐measures ANOVA) of mesenteric arteries from PMCA4KO mice. Mean ± S.E.M., *n* = 6 and 8.

Ablation of PMCA4 had no significant effect on the magnitude of isolated mesenteric arterial contraction in response to a 100 mM K^+^‐depolarizing stimulus (KPSS) (Fig. 3Ai). Contractile responses and sensitivity to NE (EC_50_ (μM): PMCA4WT = 0.90 ± 0.37, PMCA4KO = 0.75 ± 0.21) were unaffected by PMCA4 ablation (Fig. 3Aii). Ablation of PMCA4 had no significant effect on the passive properties of isolated small mesenteric arteries (internal diameter, wall thickness, cross‐sectional area) (Fig. S2).

Incubating arteries from WT mice with 10 μM ATA significantly reduced the magnitude of contraction to KPSS (Fig. 3Bi) and significantly reduced contraction and sensitivity to NE (EC_50_ (μM): WT = 0.34 ± 0.03, +ATA = 0.64 ± 0.09) (Fig. 3Bii). WT arteries incubated with 1 μM ATA also exhibited a significant reduction in contractility to KPSS (69.1 ± 3.0% to 61.0 ± 6.0%) and NE (maximum response (Rmax) = 78.6 ± 2% and 61.7 ± 4.3% vehicle/+ ATA, respectively. 10 μM ATA had no significant effect on contractility to KPSS or NE in arteries from PMCA4KO mice (Fig. 3C).

**Figure 4:**
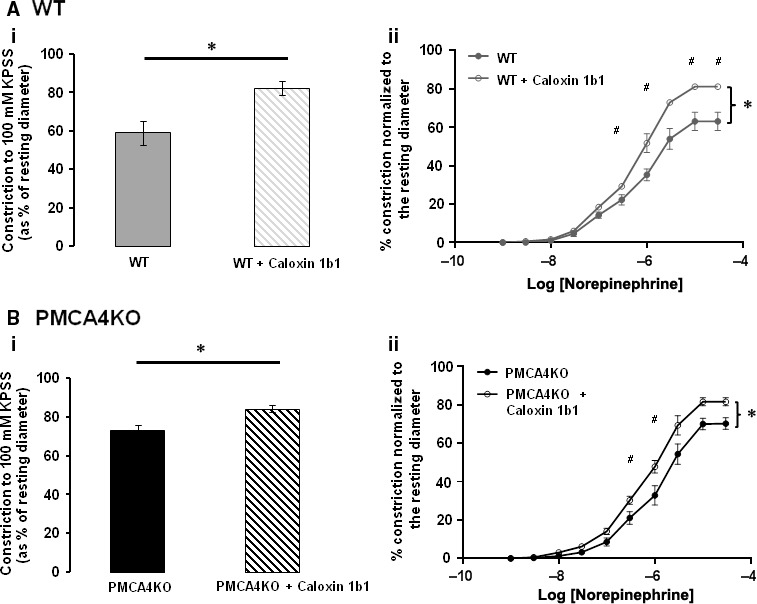
Caloxin 1b1 increases both WT and PMCA4KO arterial contractility. The magnitude of WT mesenteric arterial contraction in response to KPSS (100 mM K^+^) (**Ai**) and cumulative dose response to norepinephrine (NE) (**Aii**) was significantly augmented (*P < 0.05) following incubation with 100 μM caloxin 1b1. Mean ± S.E.M., *n* = 4 and 3. Augmentation of contractility following incubation with 100 μM caloxin 1b1 was also significant (*P < 0.05) in mesenteric arteries from PMCA4KO mice, as shown by response to KPSS (**Bi**) and cumulative dose response to NE (**Bii**). Mean ± S.E.M., *n* = 7 and 4. Magnitude of contractility (i) assessed by *t*‐test. Dose–response relationship (ii) assessed by repeated‐measures anova with Bonferroni post hoc test (^#^ *P* < 0.05 at specific log concentrations of NE).

To assess the effects of ATA, with respect to a previously studied inhibitor of PMCA4, the effects of caloxin 1b1 on mesenteric arteries were also studied on our pressure myograph setup. Caloxin 1b1 significantly increased contractility to KPSS and NE in arteries from WT mice (Fig. 4A). Caloxin 1b1 treatment also significantly increased contraction to both KPSS and NE in arteries from PMCA4KO mice (Fig. 4B).

### How may ATA mediate its action in mesenteric arteries?

**Figure 5:**
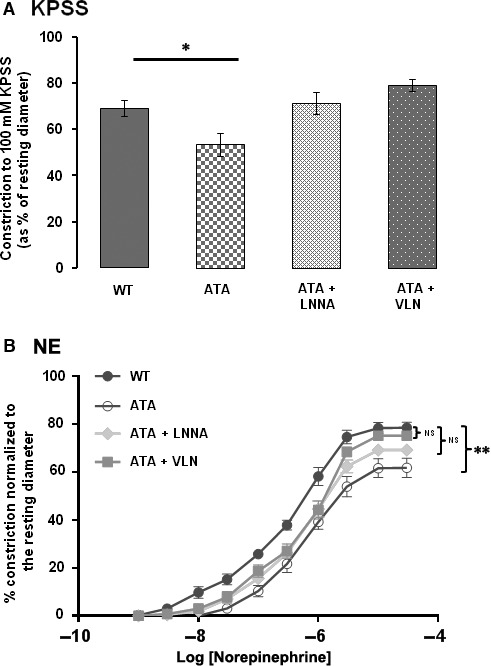
ATA mediates its effect on arterial contractility via a nNOS‐dependent mechanism. ATA significantly (*P < 0.05) reduces WT arterial contractility to KPSS (100 mM K^+^) (**A**) and norepinephrine (NE) (**B**), but no significant reduction in contractility is found following co‐incubation of arteries with ATA and the non‐specific NOS inhibitor LNNA. Mean ± S.E.M., *n* = 8 and 8. Furthermore, no significant reduction in contraction is recorded from arteries incubated with both ATA and the specific nNOS inhibitor Vinyl‐l‐NIO (VLN). Mean ± S.E.M., *n* = 5 and 8. Response to KPSS assessed by *t*‐test. Dose–response relationship assessed by repeated‐measures ANOVA.

The anticontractile effects of ATA (10 μM) on responses to KPSS and NE were abolished when arteries from WT mice were co‐incubated with ATA and the non‐specific NOS inhibitor LNNA (100 μM) (Fig. 5A and B, respectively). Incubation of arteries with the nNOS specific inhibitor VLN (10 μM) also abolished the anticontractile effects of ATA (Fig. 5). Incubation with LNNA (100 μM) alone had no effect on the contractility of mesenteric arteries obtained from PMCA4WT mice to 100 mM KPSS (in the absence of LNNA treatment = 56.3 ± 3.6%, *n* = 14; and in the presence of LNNA treatment = 60.8 ± 4.9%, *n* = 8). The same was seen for the arterial responses to NE (Rmax in the absence of LNNA = 58.8 ± 2.5%, *n* = 14; and 62.6 ± 1.7%, *n* = 8 in the presence of LNNA).

**Figure 6:**
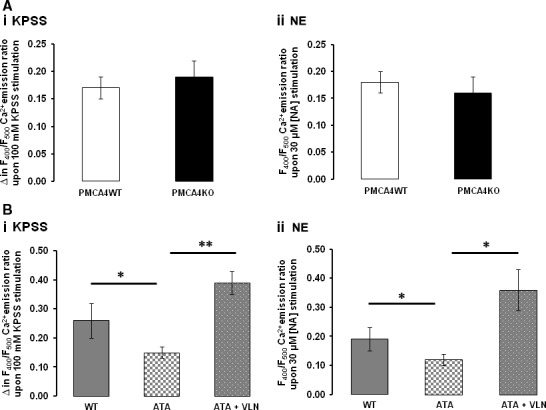
Ablation and inhibition of PMCA4 have differential effects on the concentration of arterial intracellular free calcium ([Ca^2+^]_i_) induced by contractile stimuli. Change (increase) in the F_400_/F_500_ Ca^2+^ emission ratio (representative of global arterial [Ca^2+^]_i_) from Indo‐1‐loaded mesenteric arteries does not significantly differ between vessels from PMCA4WT and PMCA4KO mice in response to KPSS (100 mM K^+^) (**Ai**) and maximal NE stimulation (**Aii**; 30 μM NE). *n* = 6 and 6. The increase in the F_400_/F_500_ Ca^2+^ emission ratio is significantly attenuated (*P < 0.05) in WT arteries incubated with ATA (10 μM) but is not significantly altered when WT arteries are co‐incubated with ATA and VLN. Response shown to KPSS (**Bi**)‐ and maximal NE stimulation (**Bii**; 30 μM NE)‐induced contraction. Mean ± S.E.M., *n* = 4 and 5.

Ablation of PMCA4 had no significant effect on the baseline Indo‐1 400 nm:500 nm fluorescence ratio (F_400_/F_500_) as a measure of [Ca^2+^]_i_ (F_400_/F_500_ = 0.65 ± 0.06 and 0.64 ± 0.06 within arteries from PMCA4WT and PMCA4KO mice, *n* = 6 and 6). Ablation of PMCA4 had no significant effect on the increase in F_400_/F_500_ in response to either 100 mM KPSS (Fig. 6Ai) or a single dose of NE (30 μM) (Fig. 6Aii). Simultaneous measurement of contraction revealed no significant effect of ablation on the contractile response to either KPSS or NE confirming our earlier findings.

Basal F_400_/F_500_ in arteries from WT mice was not significantly altered following incubation with ATA or VLN alone (F_400_/F_500_ = 0.70 ± 0.14, 0.75 ± 0.09 and 0.65 ± 0.14 for vehicle, with ATA and with VLN, respectively, *n* = 4 and 4). However, in response to 100 mM KPSS and 30 μM NE, the rise in F_400_/F_500_ was significantly attenuated in arteries with ATA compared with WT arteries alone (Fig. 6Bi and Bii, respectively). Arteries co‐incubated with ATA and VLN exhibited an increase in the rise in F_400_/F_500_ in response to both KPSS and NE stimulation, which was not significantly different to that recorded from WT arteries alone (Fig. 6B). Example trace recordings of the Indo‐1 400 nm:500 nm fluorescence ratio (F_400_/F_500_) as a measure of [Ca^2+^]_i_ are shown in Figure S3 for each group.

## Discussion

We here show, for the first time, that genetic ablation of PMCA4 in mice has no effect on either conscious peripheral BP or resistance arterial contractility to either KPSS or NE. In contrast, acute inhibition of PMCA4 with ATA, a recently characterized inhibitor of PMCA4 24, 25, reduces BP and isolated mesenteric artery contractility in response to these stimuli. The effects of ATA on conscious BP and arterial contractility are not observed in the presence of VLN, demonstrating that ATA can act *via* a nNOS‐dependent mechanism and that these effects were independent of PMCA4's role as a calcium pump, as ATA had no significant effect on global [Ca^2+^]_i_ when nNOS was inhibited.

The PMCA4KO mouse model used in this study has previously been shown to exhibit a complete lack of PMCA4 protein in testes, sperm 26 and cardiomyocytes 37. In the present study, we could detect no PMCA4 protein in isolated mesenteric VSMCs from PMCA4KO mice. In blood vessels, PMCA4 expression has previously been shown to be predominant in VSM but not in vascular endothelium 15, 16, and we have confirmed this in aortic sections. Technical difficulties in sectioning small mouse mesenteric arteries, and particularly in preserving the endothelial layer intact, prevented us from confirming localization in these arteries, and we accept this is a limitation. Previous work has shown that global ablation of PMCA4 has no significant effect on the expression of other Ca^2+^ handling proteins in the heart 26, 37 or in bladder smooth muscle 38, and herein we report that arterial PMCA1 and NCX1 protein expression is not significantly affected by loss of PMCA4. Hence, other Ca^2+^ pumps and channels appear to not compensate for the loss of PMCA4. Our analysis of intact aortic segments from WT mice by immunohistochemistry showed that PMCA4 is principally localized to VSMCs with little being detected in endothelial cells, observations consistent with previous reports 14, 16. It remains a challenge to definitively quantify PMCA4 expression in resistance arteries, or cells derived from such vessels, but this remains an objective in directly supporting functional studies.

Previous studies have shown unconscious BP is elevated in mice overexpressing PMCA4 in arterial VSMCs 14, 17. For the first time, we show that global ablation of PMCA4 had no significant effect on conscious peripheral BP as measured by tail cuff in conscious mice. Also, previous studies have shown that arterial contractility to vasoactive stimuli is increased following overexpression of PMCA4 14, 17, whereas herein we show that complete ablation of PMCA4 protein had no effect on the contractility of isolated mesenteric resistance arteries to either KPSS‐mediated depolarization (100 mM K^+^) or NE. Hence, the functional consequence of global PMCA4 ablation is not directly opposite to reported effects when PMCA4 is overexpressed in VSM 14, 17.

As the reason for this remains unclear, we sought to clarify the role of PMCA4 in BP regulation by acutely inhibiting PMCA4 with ATA, a recently identified and validated specific inhibitor of PMCA4 24. We have previously shown that ATA at 1 μM inhibits 80% of PMCA4 activity in an *in vitro* assay and at 10 μM fully inhibits PMCA4 activity in cardiomyocytes 24; hence, these concentrations were selected for the *in vitro* functional studies reported here. We also selected a comparable dose *in vivo* of 5 mg/kg, and at this same dose, ATA was shown to significantly attenuate pressure overload‐induced cardiac hypertrophy and it reversed established cardiac hypertrophy 25. Such doses are well below the threshold for other effects of ATA to be promoted, as previously described 7, 24. The half‐life of ATA *in vivo* has not been precisely defined; however, at 35 mg/kg it had a significant biological effect for up to 2 hrs in rats 39.

We here present that a single dose of ATA (5 mg/kg) significantly reduced systolic and diastolic conscious BP as measured 90 min. after i.p. injection, an observation consistent with both 1 and 10 μM ATA significantly reducing PMCA4WT arteries contractility to a depolarizing and adrenergic stimulus. Importantly, ATA was found to have no significant effect on BP or on arterial contractility of mesenteric arteries from PMCA4KO mice, thus displaying a specificity of action and supporting our previous findings that ATA has little effect on other calcium handling proteins 28.

This study reports, for the first time, the effects of ATA on arterial function and compares these with the effects of caloxins which have previously been the only other available inhibitors of PMCA4 18, 19, 22, 27, 40, 41. In contrast to our finding that ATA *reduces* contractility of mesenteric vessels isolated from WT mice, but not PMCA4KO mice, caloxin 1b1 increased the contractility of mesenteric arteries to both a depolarizing stimulus and to NE. Importantly, caloxin 1b1 increased the contractility of arteries isolated from both PMCA4WT and PMCA4KO mice, suggesting that it can modulate contractility *via* effects which are independent of the presence of PMCA4 protein. Whilst this may explain, at least in part, the differing effects of ATA and caloxins on arterial contractility, differences in the mechanism of action of the two compounds may clearly contribute. It is worth noting that previous studies reporting that caloxin peptides increased arterial contractility utilized aortic and coronary vessels 18, 19, vessels which contribute very little to total peripheral arterial resistance and in turn BP. Therefore, in treating mesenteric vessels with ATA the current study informs on the role of PMCA4 in relation to BP regulation.

Further, we sought to understand how inhibition of PMCA4 can reduce arterial contractility and BP. Previous PMCA4 overexpression studies which have reported increased vascular contraction have attributed this observation to the negative regulatory effects of PMCA4 on nNOS 14, 17, an effect which has been well characterized in the heart and in *in vitro* cellular systems 20, 21, 23. In WT mice, we showed that the effects of acute PMCA4 inhibition with ATA on BP and isolated arterial contractility were not observed in the presence of inhibitors of nNOS, suggesting these effects of ATA are also *via* a nNOS‐dependent mechanism(s). nNOS expression and activity have previously been demonstrated in the vasculature including in mesenteric arteries 17, 42, 43, 44, 45. Previous studies have demonstrated the importance of nNOS on arterial tone, with changes in/ablation of nNOS expression 14, or with overexpression of PMCA4 as a regulator of nNOS activity 17 or in disease states such as hypertension 42, 45. In contrast, we found no significant effect of inhibition of nNOS with VLN or NOS with LNNA alone on BP or on arterial contractility, respectively. The lack of significant effect of LNNA on agonist‐induced constrictions of mesenteric arteries from WT mice is consistent with previous reports by our group and others 30, 46. Taken together, this suggests that the role played by nNOS in regulation of these parameters gains importance in states whereby there are changes in the level of nNOS expression or in the regulation of its activity. The nNOS‐dependent effects of ATA we observed are consistent with an increase in nNOS activity as a result of removal of the negative regulatory effects of PMCA4 on nNOS 14, 17. Whilst we acknowledge that we did not investigate whether ATA modulates nNOS expression, we think this unlikely given the acute application.

Activation of nNOS has been shown to be *via* Ca^2+^‐dependent activation of calmodulin and subsequent Ca^2+^/calmodulin interaction with nNOS 21, 47. In the heart, PMCA4 physically tethers nNOS 21, 37, and by mediating expulsion of Ca^2+^ from a microdomain at the plasma membrane, it may reduce the ability of the associated synthase to be activated 37. This modulation appears to be due to effects of PMCA4 on local subcellular [Ca^2+^]_i_ 20, 48, 50 and not directly on global [Ca^2+^]_i_. Such a mechanism of action remains to be tested in resistance artery VSMCs; however, although ATA reduced increases in global [Ca^2+^]_i_ to stimulation in isolated mesenteric arteries, this effect was prevented by LNNA or VLN, suggesting that its effects were due to nitric oxide *per se* rather than being PMCA‐mediated. Indeed, it is well‐established that nitric oxide, *via* its second messengers cGMP and PKG, can reduce [Ca^2+^]_i_ in vascular smooth muscle by mechanisms which include decreased Ca^2+^ entry and reduced release from the sarcoplasmic reticulum 51, 52, 53. This observation is consistent with the notion that inhibiting PMCA4 has no effects on global [Ca^2+^]_i_ and is consistent with the regulative mechanism in the heart, whereby PMCA4 regulates nNOS activity by physical tethering and regulation of subcellular Ca^2+^. Indeed, ablation of PMCA4 had no effect on global [Ca^2+^]_i._ Activation of nNOS is Ca^2+^‐dependent 21, 54. Activation of PMCAs is dependent on Ca^2+^/calmodulin binding with increases in [Ca^2+^]_i_ causing Ca^2+^/calmodulin binding to the autoinhibitory domain of PMCA which, in turn, causes a conformational change and release of the autoinhibitory effect 55. Isolated arteries used in the present study did not develop intrinsic tone. Taken together, this likely underpins the lack of effect of ATA on resting tissues.

The relevance of PMCA4 in the scaffolding of regulators to subcellular domains in recent discussions 56 is of key importance and particular interest. This is vital to the actions of PMCA4 as demonstrated by different functional effects (on cell cycle progression) being presented between our PMCA4KO model, in which there is a complete lack of PMCA4 protein 26, and an alternate model, in which there is mutant, non‐functioning PMCA4 present 56, 57. This is consistent with an important scaffolding role for PMCA4. Indeed, the lack of any effect of global ablation of PMCA4 on BP or on arterial contractility we observed in the present study is likely to be underpinned by this. This contrasts with effects of PMCA4 inhibition (in the present study) and PMCA4 overexpression 14, 17 where the physical interaction is present and effects are seen.

We propose that arterial PMCA1 or NCX1 does not compensate for ablation of PMCA4 in PMCA4KO mice. However, it remains inconclusive whether nNOS expression and/or function may be up‐regulated in resistance arteries in association with PMCA4 ablation. We have previously shown that global ablation of PMCA4 does not affect the total protein level of nNOS in the heart, but rather that cardiomyocyte nNOS was localized more in the cytoplasm and not at the cells’ plasma membrane 37. Although the possibility of relocalization of active nNOS occurring in resistance artery VSMCs of PMCA4KO mice remains to be determined, this concept could contribute to explaining how BP is regulated with chronic loss of PMCA4. That PMCA4 maintains the spatial and functional integrity of a signalling complex including nNOS in a defined plasma membrane microdomain 14, 37, 58 is well supported, but that its complete absence may cause disruption of the complex with nNOS being relocated to the cytosol remains to be fully elucidated. This would further promote an important role for PMCA4 as a scaffolding molecule in arterial tissue as has recently been discussed 51.

**Figure 7:**
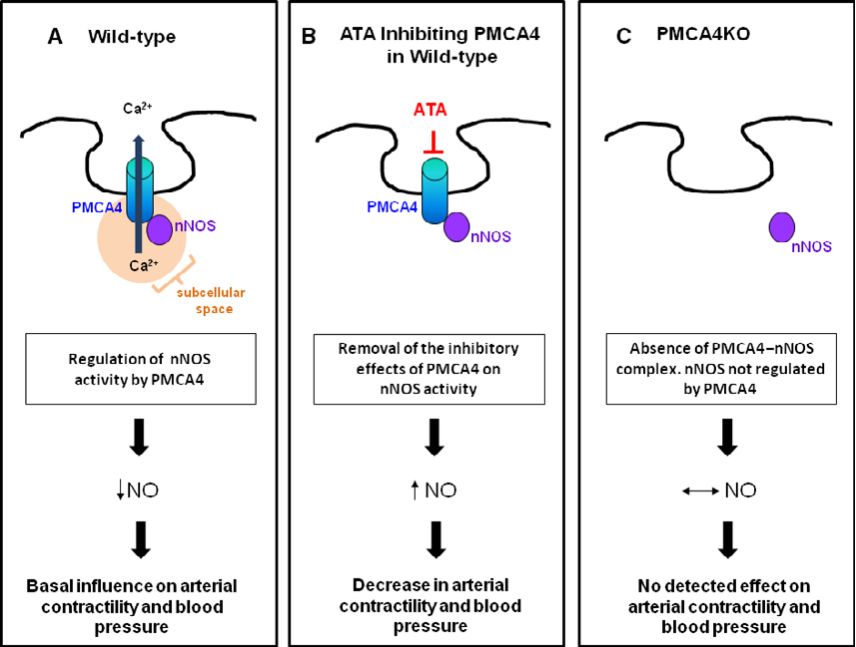
Schematic representing the proposed mechanisms underpinning the effects of how PMCA4 contributes to regulating arterial contractility *via* nNOS. In vascular smooth muscle cells, PMCA4 is principally localized in invaginations of the membrane. (**A)** In wild‐type animals, PMCA4 physically tethers nNOS and negatively regulates its activity, most likely due to effects on local subcellular [Ca^2+^]_i_. This modulates basal levels of nitric oxide which influences arterial contractility and blood pressure. (**B)** Inhibition of PMCA4 by ATA significantly reduces the calcium pumping activity of PMCA4 even though the physical PMCA4–nNOS complex is retained. Consequently, the inhibitory effect of PMCA4 on nNOS is lost resulting in an increase in nNOS activity and nitric oxide production which subsequently reduces arterial contractility and blood pressure. (**C)** Global ablation of PMCA4 (PMCA4KO) results in nNOS no longer being physically tethered to PMCA4, and its activity is no longer regulated by changes in local subcellular [Ca^2+^]_i._ No effects on the level of nNOS, nitric oxide, arterial contractility or blood pressure are seen. Whether the precise cellular localization of nNOS remains a factor in the regulation of arterial contractility and blood pressure in PMCA4KO animals remains to be determined.

In summary, we have shown using a novel specific inhibitor against PMCA4, ATA, that inhibition of PMCA4 reduces conscious peripheral BP and isolated mesenteric arterial contractility *via* a PMCA4–nNOS‐dependent mechanism. We propose PMCA4 contributes to regulating BP *via* a nitric oxide‐dependent signalling pathway rather than a direct effect on global [Ca^2+^]_i_‐mediated VSMC contraction and highlight the importance of PMCA4 as a scaffolding molecule in resistance vessels (see Fig. 7 for a schematic summarizing the proposed mechanisms underpinning the effects of how PMCA4 contributes to the regulation of arterial contractility *via* nNOS). Herein we show specificity of action of ATA for PMCA4, which contrasts with our findings using a commercial PMCA4 inhibitor, caloxin 1b1. Further characterization of ATA is required, and whilst we cannot propose ATA *per se* as a therapeutic agent, we do propose its use as an important experimental tool to further define the relationship between PMCA4 and BP. Understanding the molecular role of PMCA4 remains important for future development of novel BP lower strategies, of which there is increasing clinical need in an ageing population and with increasing intolerance to current interventions.

## Author contributions

S Lewis, R Little, S Prehar, F Baudoin performed the research; S Lewis and R Little analysed the data; C Austin, EJ Cartwright and L Neyses designed the research study; L Neyses, EJ Cartwright and C Austin contributed to acquiring funding; EJ Cartwright contributed essential tools; R Little, S Lewis, EJ Cartwright and C Austin contributed to writing the manuscript.

## Conflicts of interest

The authors declare no conflict of interest.

## Supporting information


**Figure S1** Differential effect of ablation and inhibition of PMCA4 on basal conscious blood pressure.

**Figure S2** Passive properties of mesenteric arteries from PMCA4WT and PMCA4KO mice are not significantly different.

**Figure S3** Examples of experimental trace recordings for each mouse genotype and pharmacologically treated group.
