# Supplementary figures and images for: Acute inhibition of PMCA4, but not global ablation, reduces blood pressure and arterial contractility via a nNOS‐dependent mechanism
Source: J Cell Mol Med. 2017 Nov 30;22(2):861–72. doi: 10.1111/jcmm.13371 (PMC5783868; doi:10.1111/jcmm.13371)

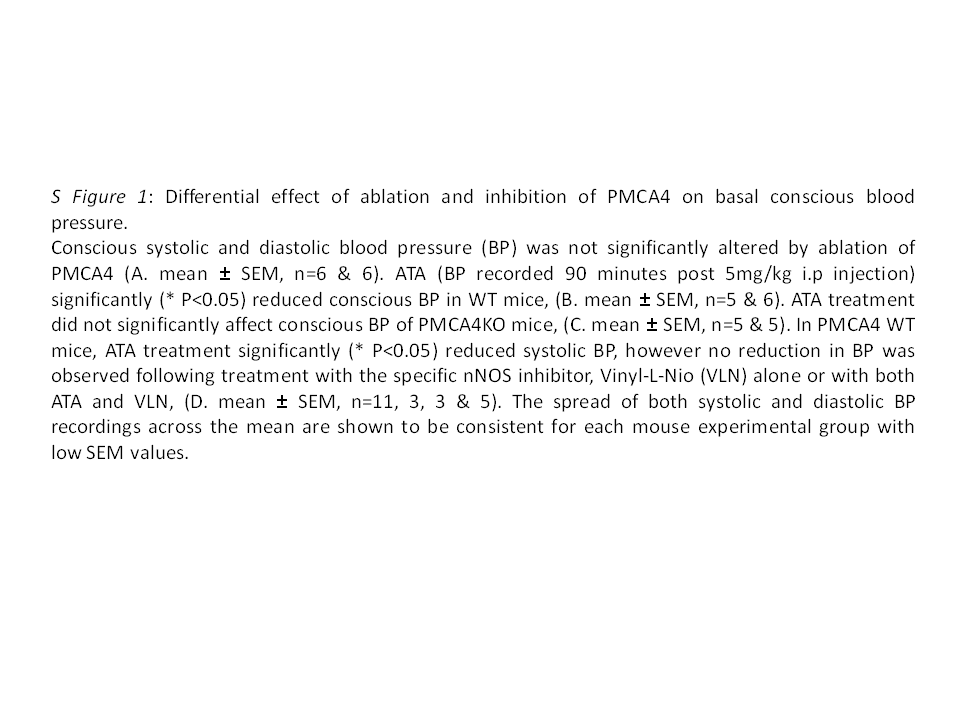

Supplement: Supplementary file 1 — Figure S1 Differential effect of ablation and inhibition of PMCA4 on basal conscious blood pressure. [file JCMM-22-861-s001.zip › Legend_SFigure1.PNG]

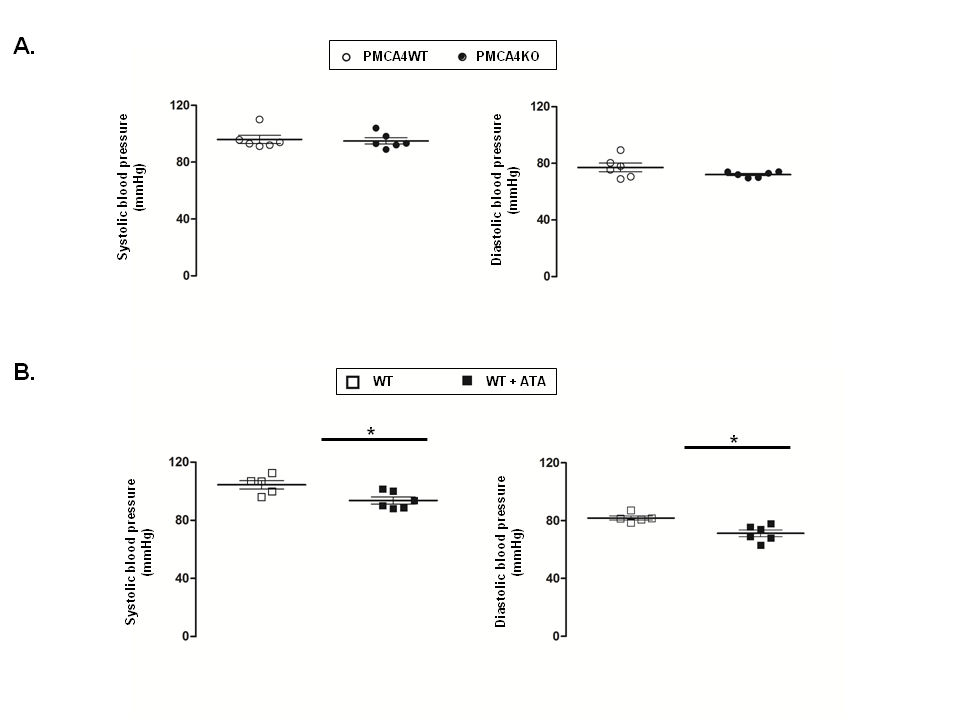

Supplement: Supplementary file 1 — Figure S1 Differential effect of ablation and inhibition of PMCA4 on basal conscious blood pressure. [file JCMM-22-861-s001.zip › Supplementary.SFigure1A,B_JCMM13371.PNG]

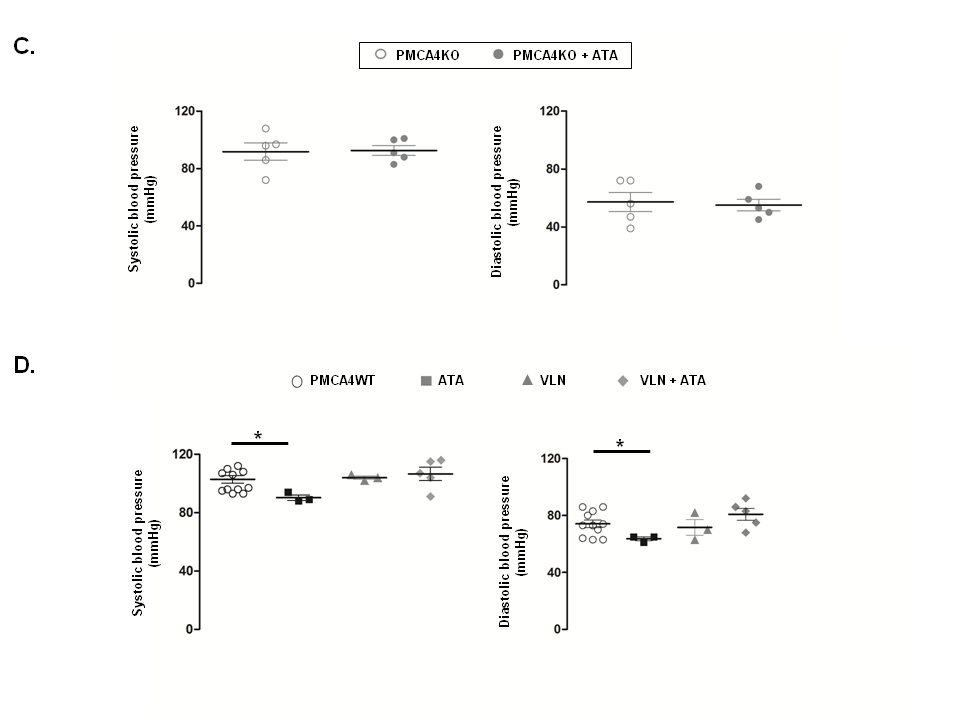

Supplement: Supplementary file 1 — Figure S1 Differential effect of ablation and inhibition of PMCA4 on basal conscious blood pressure. [file JCMM-22-861-s001.zip › Supplementary.SFigure1C,D_JCMM13371.PNG]

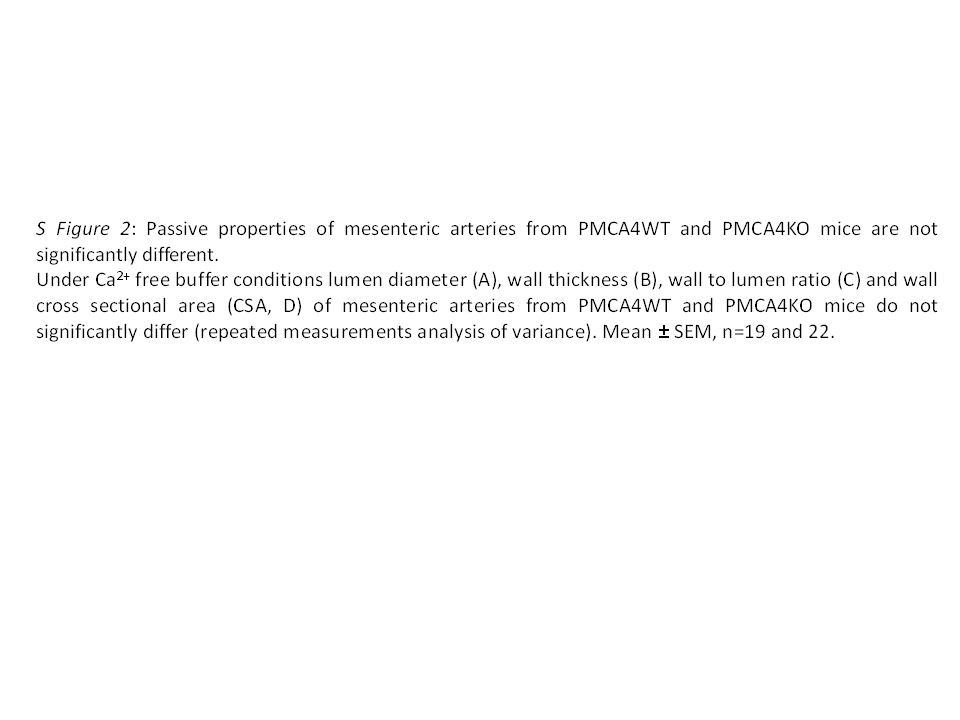

Supplement: Supplementary file 2 — Figure S2 Passive properties of mesenteric arteries from PMCA4WT and PMCA4KO mice are not significantly different. [file JCMM-22-861-s002.zip › Legend_SFigure2.PNG]

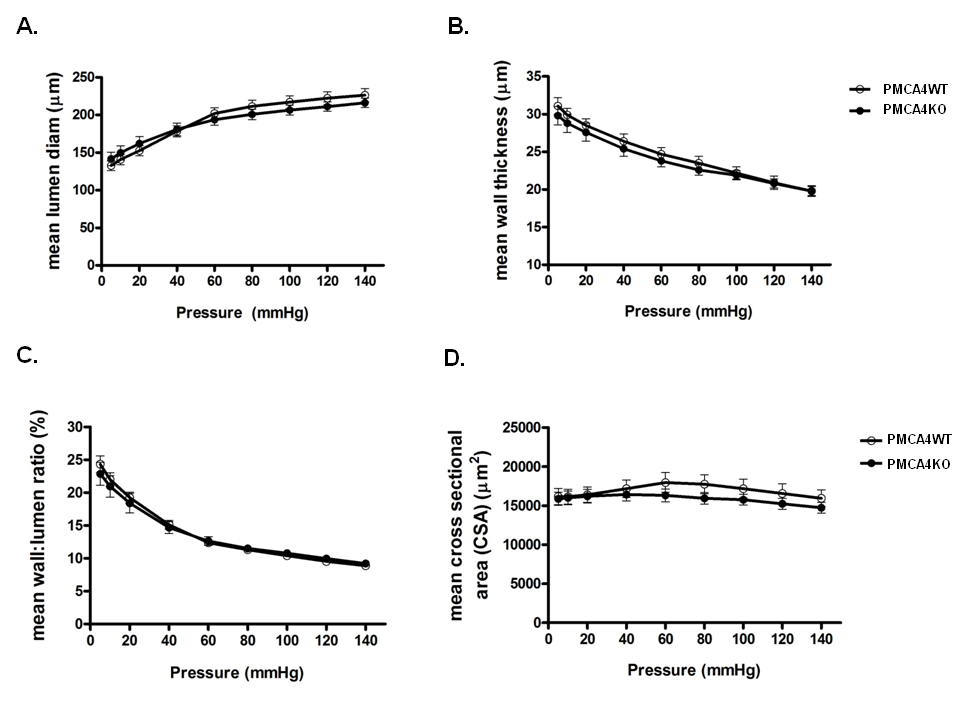

Supplement: Supplementary file 2 — Figure S2 Passive properties of mesenteric arteries from PMCA4WT and PMCA4KO mice are not significantly different. [file JCMM-22-861-s002.zip › Supplementary.SFigure2_JCMM13371.PNG]

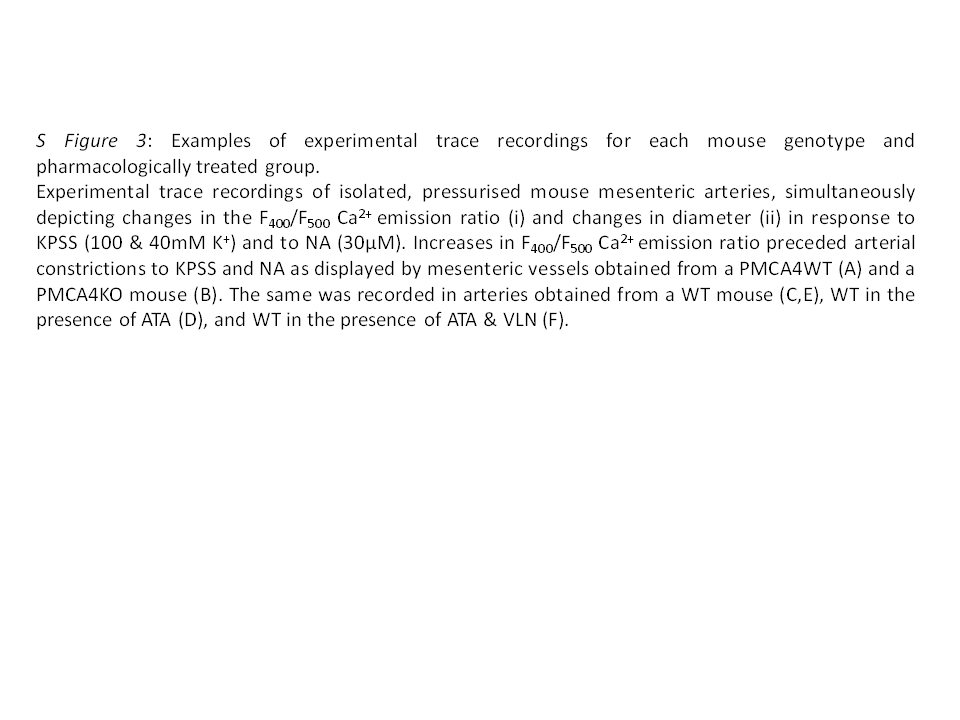

Supplement: Supplementary file 3 — Figure S3 Examples of experimental trace recordings for each mouse genotype and pharmacologically treated group. [file JCMM-22-861-s003.zip › Legend.SFigure3.PNG]

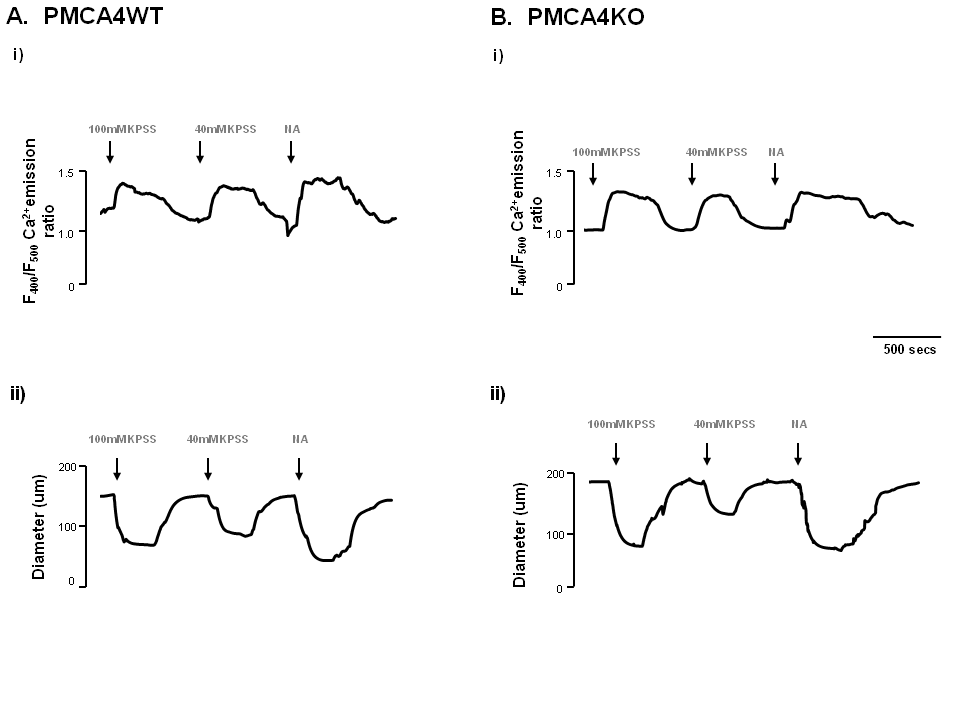

Supplement: Supplementary file 3 — Figure S3 Examples of experimental trace recordings for each mouse genotype and pharmacologically treated group. [file JCMM-22-861-s003.zip › Supplementary.SFigure3A,B_JCMM13371.PNG]

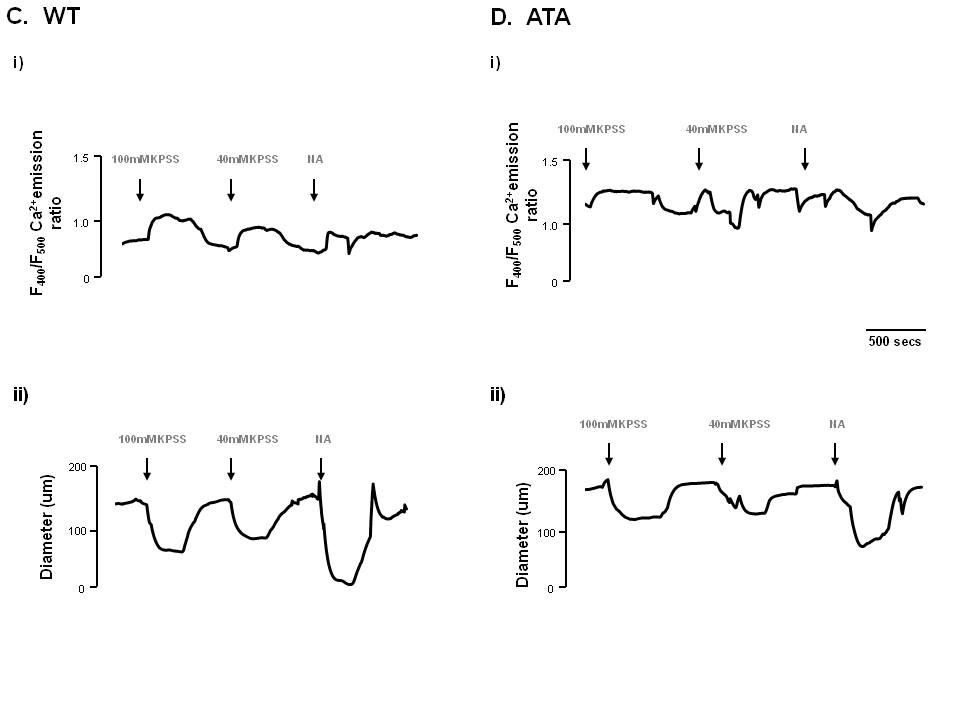

Supplement: Supplementary file 3 — Figure S3 Examples of experimental trace recordings for each mouse genotype and pharmacologically treated group. [file JCMM-22-861-s003.zip › Supplementary.SFigure3C,D_JCMM133.PNG]

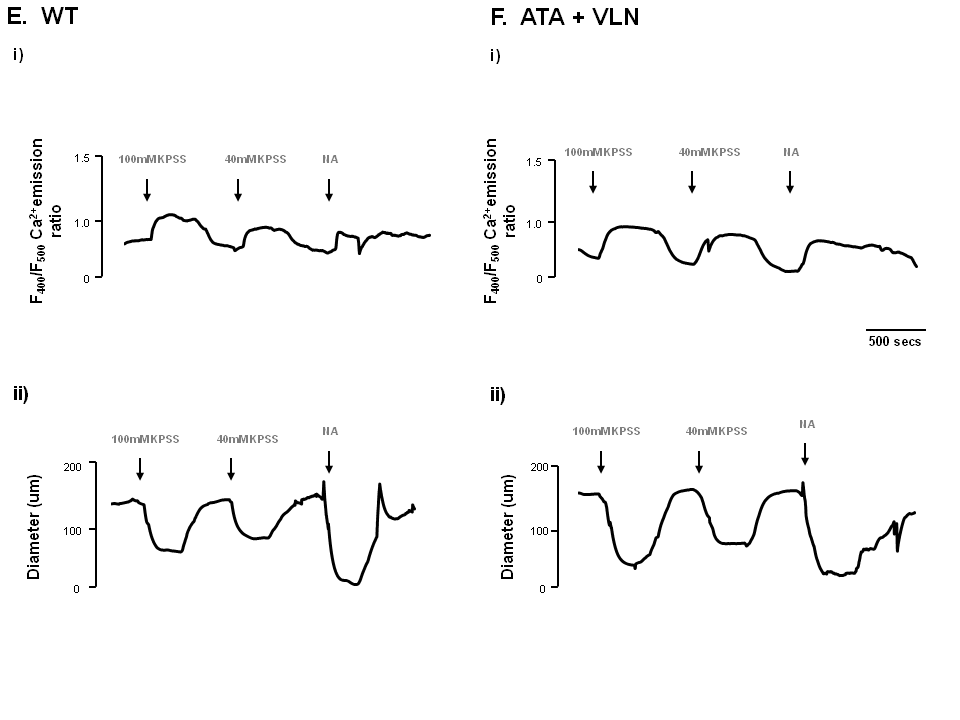

Supplement: Supplementary file 3 — Figure S3 Examples of experimental trace recordings for each mouse genotype and pharmacologically treated group. [file JCMM-22-861-s003.zip › Supplementary.SFigure3E,F_JCMM133.PNG]
